# A Novel PAMAM G3 Dendrimer-Based Foam with Polyether Polyol and Castor Oil Components as Drug Delivery System into Cancer and Normal Cells

**DOI:** 10.3390/ma17163905

**Published:** 2024-08-07

**Authors:** Magdalena Zaręba, Elżbieta Chmiel-Szukiewicz, Łukasz Uram, Justyna Noga, Magdalena Rzepna, Stanisław Wołowiec

**Affiliations:** 1 The Faculty of Chemistry, Rzeszow University of Technology, 35-959 Rzeszow, Poland; magzar@prz.edu.pl (M.Z.); szukela@prz.edu.pl (E.C.-S.); luram@prz.edu.pl (Ł.U.); justynanoga258@gmail.com (J.N.); 2 Centre for Radiation Research and Technology, Institute of Nuclear Chemistry and Technology, 03-195 Warsaw, Poland; m.rzepna@ichtj.waw.pl; 3 Medical College, University of Rzeszow, 1a Warzywna Street, 35-310 Rzeszow, Poland

**Keywords:** PAMAM G3-based foam, polyether polyol, castor oil, drug encapsulation, SCC-15 and HaCaT cells, immobilization, doxorubicin

## Abstract

One of the intensively developed tools for cancer therapy is drug-releasing matrices. Polyamidoamine dendrimers (PAMAM) are commonly used as nanoparticles to increase the solubility, stability and retention of drugs in the human body. Most often, drugs are encapsulated in PAMAM cavities or covalently attached to their surface. However, there are no data on the use of PAMAM dendrimers as a component of porous matrices based on polyurethane foams for the controlled release of drugs and biologically active substances. Therefore, in this work, porous materials based on polyurethane foam with incorporated third-generation poly(amidoamine) dendrimers (PAMAM G3) were synthesized and characterized. Density, water uptake and morphology of foams were examined with SEM and XPS. The PAMAM was liquefied with polyether polyol (G441) and reacted with polymeric 4,4′-diphenylmethane diisocyanate (pMDI) in the presence of silicone, water and a catalyst to obtain foam (PF1). In selected compositions, the castor oil was added (PF2). Analogs without PAMAM G3 were also synthesized (F1 and F2, respectively). An SEM analysis of foams showed that they are composed of thin ribs/walls forming an interconnected network containing hollow bubbles/pores and showing some irregularities in the structure. Foam from a G3:G441:CO (PF2) composition is characterized by a more regular structure than the foam from the composition without castor oil. The encapsulation efficiency of drugs determined by the XPS method shows that it varies depending on the matrix and the drug and ranges from several to a dozen mass percent. In vitro biological studies with direct contact and extract assays indicated that the F2 matrix was highly biocompatible. Significant toxicity of dendrimeric matrices PF1 and PF2 containing 50% of PAMAM G3 was higher against human squamous carcinoma cells than human immortalized keratinocytes. The ability of the matrices to immobilize drugs was demonstrated in the example of perspective (Nimesulide, 8-Methoxypsolarene) or approved anticancer drugs (Doxorubicin—DOX, 5-Aminolevulinic acid). Release into the culture medium and penetration of DOX into the tested SCC-15 and HaCaT cells were also proved. The results show that further modification of the obtained matrices may lead to their use as drug delivery systems, e.g., for anticancer therapy.

## 1. Introduction

PAMAM dendrimers belong to a new class of polymers characterized by a hyperbranched structure, nanometric size and the ability to control structural parameters at the stage of their synthesis. This means that they have a specific size, shape and molecular weight and are monodisperse [1,2,3]. Additionally, it is possible to modify their surface amino groups, which opens up unlimited possibilities for various derivatives depending on the purpose [4,5,6]. The availability of PAMAM dendrimers and the possibility of designing the final macromolecule determines the wide range of their applications [7,8,9]. The polypeptide interior and nanoscale dimensions, which give them the name “artificial proteins”, as well as biodegradability and biocompatibility, imply particular the use of PAMAMs in the transport of drugs [10,11,12]. The use of PAMAM dendrimers as drug nano-vehicles has many advantages, including increasing the solubility of hydrophobic drugs and enabling their controlled release, obtaining higher stability and extending their release time [13,14,15]. PAMAM can bind the drugs in two ways: conjugation via covalent bonds with surface groups or encapsulation in the open space between dendrons through hydrogen and electrostatic interactions. Encapsulated drugs gain protection among the branches of the dendrimer, which prevents its degradation and guarantees higher effectiveness [16,17]. Properties of PAMAM dendrimers enabling the transport of foreign molecules make them currently one of the most frequently studied drug nanocarriers, mainly in targeted anticancer therapy or transdermal transport [18,19,20]. In addition to the widespread use of PAMAM dendrimers as independent drug delivery units, there are also a few reports of their use as matrix elements for skin tissue engineering, e.g., gelatin/PAMAM scaffolds [21], PAMAM/gelatin hydrogel [22], PAMAM/pluronic F127 nanofilm [23] or highly cross-linked (PAH/PAMAM)15-CaCO_3_ composite film [24]. 

Other polymer matrices with biomedical applications are solid polymer foams [25,26]. These include biodegradable porous starch foam (BPSF) used as a carrier in drug delivery to improve the dissolution and enhance the oral bioavailability of poorly water-soluble drugs [27,28], cellulose nanofiber-based foams (CNF) used as sustained drug delivery system [29] and polyurethane-based shape memory foams (SMP) used in the field of self-deployable medical devices [30]. If solid polymer foams can be used for biomedical purposes, they must be biocompatible and biodegradable [31,32]. Therefore, biologically or synthetically derived biomaterials are used to obtain them; most often, these are natural oil-based polyols [33,34] or glycerin-based polyols [35,36,37]. Castor oil (CO) is a vegetable oil consisting mainly of ricinoleic acid ester. It has a hydroxyl group, which makes it ideal for the production of polyurethane foams [38]. It can also be modified with other compounds, such as glycerol [39] or diethanolamine and triethanolamine [40]. This results in castor oil polyols, which, when used to synthesize PU, can improve their properties [41]. In addition, CO has antifungal and antibacterial properties and prevents inflammation [42,43]. Its presence may reduce the likelihood of matrix contamination and microbial growth after implantation. Moreover, most cancers are accompanied by inflammation within the tumor, and its reduction usually leads to an increase in the success of the therapy [44].

In the treatment of cancer, it is often seen that therapy also damages normal cells. It is, therefore, important to develop an effective system that delivers the drug only to the affected areas. One construct that can be used for this purpose is a polymer matrix, for example, in the form of foams [26,45]. Polyurethane foams have a porous structure that allows medication to be placed inside the foam. They are also flexible, which means they can be molded to fit the area where they are to be placed. Studies have shown that polyurethane foams have low toxicity and can be used for controlled drug release [46,47]. In addition, appropriately constructed foams can serve as scaffolds to aid tissue recovery and regeneration following illness [48]. One of the drugs that can be incorporated into the foams is doxorubicin (DOX), an anthracycline antibiotic commonly used to treat solid tumors and hematological malignancies, such as lymphoma, lung or breast cancer [49]. DOX binds to double-stranded DNA, which inhibits cell division and causes cell death. According to the study, polyurethane foams synthesized from lysine diisocyanate (LDI) and glycerol, containing doxorubicin gradually release the drug to the outside but also stabilize its fluorescence, making it easier to image [50]. Also, PAMAMs have been used successfully as doxorubicin carriers. DOX was encapsulated or conjugated via various types of bonds to native or modified (including those targeted to specific cancer types) PAMAM dendrimers. It has been repeatedly demonstrated that dendrimers and doxorubicin can create excellent platforms for drug release and targeted chemotherapy of cancer cells [51,52,53].

Taking into account the mentioned advantages of PAMAM dendrimers as nanocarriers, in this study, we investigated the possibility of using PAMAM G3 dendrimers as components of polyurethane matrix to improve immobilization and the release of potential anticancer drugs. Despite the comprehensive literature on PAMAM dendrimers, there are no reports on foams with their participation. The exception is Chinese patents CN101831046 (A) and CN101831046 (B) [54], in which first-, second- and third-generation PAMAM dendrimers have been used in small amounts, at levels of a few percent, as crosslinking agents for thermally insulating polyurethane foams. Therefore, to fill this research gap, we present a method for synthesizing porous materials, i.e., foams, using PAMAM dendrimer as a polyamine compound. Moreover, the biological activity and cytotoxicity of obtained matrices with or without doxorubicin were estimated on human squamous cell carcinoma SCC-15 cells and comparatively on human immortalized keratinocytes (HaCaTs)—models of normal cells. 

## 2. Materials and Methods

### 2.1. Materials

The polyoxyalkylene triol (Rokopol^®^G441) was supplied by PCC Rokita (Brzeg Dolny, Poland). 4,4′-Diphenylmethane diisocyanate (mixture of di- and tri-isocyanates) for synthesis, (pMDI, Merck Life Science, Darmstadt, Germany); Triethylamine (TEA, 99.5%, Chempur, Piekary Śląskie, Poland); Silicone PU-8580 (Silibase, Jiande China); Castor oil (Merck Life Science, Darmstadt, Germany); Dimethyl sulfoxide (DMSO, Sigma-Aldrich, Saint Louis, MO, USA); Ethanol (Supelco, Bellefonte, PA, USA); Dulbecco′s Phosphate-Buffered Saline (PBS, Sigma-Aldrich, Saint Louis, MO, USA); Nimesulide (NMS, Sigma-Aldrich, Saint Louis, MO, USA); Aminolevulinic acid hydrochloride (ALA, Pol-Aura, Zawroty, Poland); Doxorubicin hydrochloride (DOX, Pol-Aura, Zawroty, Poland); 8-Methoxypsoralen (MOP, Sigma-Aldrich, Saint Louis, MO, USA) were all used as received. PAMAM G3 dendrimer was obtained according to Tomalia et al. [2] and purified according to Esfand and Tomalia [8], as we described earlier [55]. 

Human squamous carcinoma cells (SCC-15 line), penicillin and streptomycin were purchased from American Type Culture Collection (ATCC, Manassas, VA, USA). Human immortalized keratinocytes (HaCaT line) were from Cell Lines Service (Eppelheim, Germany). Dulbecco’s modified Eagle’s medium F12 (DMEM-F12), Dulbecco’s modified Eagle’s medium (DMEM) and fetal bovine serum (FBS) were obtained from Corning (New York, NY, USA). Phosphate-buffered saline (PBS) with and without magnesium and calcium ions, crystal violet, neutral red solution, 0.4% trypan blue solution, sterile syringe filters 0.22 μm, neutral red solution, XTT sodium salt, phenazinemethosulfate (PMS) were provided by Sigma–Aldrich (St. Louis, MO, USA). Trypsin–EDTA solution was delivered by Gibco Thermofischer Scientific (Waltham, MA, USA). Cell culture flasks and other sterile plastics were from Corning Incorporated (Corning, NY, USA), and 6-well and 96-well plates were from Nunc (Roskilde, Denmark).

### 2.2. PAMAM G3 Dendrimer Foaming Procedure

To a 150 cm^3^ polypropylene cup, we added an appropriate amount of PAMAM G3, glycerin-based polyether polyol (with hydroxyl number value within the range of 330–360 mg KOH/g and dynamic viscosity 250–310 mPas at temp. 25 °C) and castor oil if it was included in the assumed composition (Table 1, foam number 5 and 12, respectively). The mixtures were heated to 50 °C and mixed until a homogenous mixture was obtained and left for at least 24 h at room temperature. After this time, the appropriate amount of silicone and water were added and mixed at a temperature of approximately 45 °C. After cooling the mixture to room temperature, the appropriate amount of pMDI (Equation (1)) and TEA were added and mixed intensively until creaming began. Foams 5 and 12, marked as PF1 and PF2, were seasoned for a week at room temperature.

(1) $$m_{NCO}=\left[\left(42\cdot \frac{m_{p}}{p_{NCO}}\right)\cdot \left(f\cdot \frac{100}{M}+\frac{p_{H_{2}O}}{18}\right)\right]\cdot k$$

where m_p_ is mass of polyol/castor oil/dendrimer agent used for foaming [g], f—functionality of the polyol/castor oil/dendrimer agent, p_NCO_—content of NCO groups in pMDI [wt%], pH_2_O—H_2_O content in relation to the weight of the polyol used agent [wt%], M—molar mass of the polyol, 42—molar mass of the NCO group, 18—molar mass of water.

### 2.3. Analytical Methods

The apparent density of foams PF1 and PF2 (the ratio of foam mass to sample volume) was determined for cubic samples according to the standard UNE EN ISO 845:2010 [56]. The water absorption test was performed in accordance with the standard [57] by immersing the samples in distilled water for 5 min, 1 h, 3 h and 24 h. Water absorption in vol % was calculated from Equation (2):

(2) $$\%W_{A}=\frac{m_{2}-m_{1}}{V_{0}\times d_{W}}\times 100\%$$

where m_1_, m_2_—mass of the sample before and after immersion in distilled water, respectively (g), V_0_—volume of the sample before immersion in distilled water (cm^3^), d_W_—density of water (d_W_ = 1 g/cm^3^). 

**SEM**

The study of the structure and morphology was carried out using a scanning electron microscope (JEOL, type JSM-6490 LV, Tokyo, Japan). During the measurements, an accelerating voltage of approx 20 kV and secondary electron detection (SEI) were used. The samples were covered with a layer of gold with a thickness of approx. 10 nm using a JEOL JFC-1300 gold sputtering machine (JEOL, Tokyo, Japan) and placed in an electron microscope chamber, then surface analyses of material samples within several micro-areas were performed.

**XPS**

X-ray photoelectron spectroscopy (XPS) studies were performed using the multi-chamber UHV system (PREVAC). Spectra were collected using hemispherical Scienta R4000 electron analyser. Scienta SAX-100 X-ray source (Al Kα, 1486.6 eV, 0.8 eV band) equipped with the XM 650 X-Ray Monochromator (0.2 eV band) were used as complementary equipment. The pass energy of the analyser was set to 200 eV for survey spectra (with 500 meV steps) and 50 eV for regions (high-resolution spectra) Ni2p, O1s, Si2p, Al2p and C1s (with 50–100 meV step). The base pressure in the analysis chamber was 5 × 10^−9^ mbar. During the spectra collection, it was not higher than 3 × 10^−8^ mbar. 

**Radiation sterilization**

Samples were irradiated with a 10 MeV electron beam generated in a linear electron accelerator (Elektronika 10/10) to a dose of 25 kGy at the Institute of Nuclear Chemistry and Technology (Warsaw, Poland). Irradiation treatments were conducted at dry ice temperature and in an air atmosphere. Dosimetry was carried out using a graphite calorimeter according to ISO/ASTM 51631:2020 [58].

### 2.4. Drug Immobilization

#### 2.4.1. Drug Solutions

Drug solutions for immobilization were prepared by dissolving the appropriate amount of nimesulide (NMS) and 8-methoxypsoralen (MOP) in a DMSO:PBS mixture (in a volume ratio of 1:9), doxorubicin hydrochloride (DOX) in water, 5-aminolevulinic acid hydrochloride (ALA) in a DMSO/ethanol mixture (in a volume ratio of 1:9) to obtain 1 mM concentration.

#### 2.4.2. Drug Immobilization for XPS Analysis

Samples with dimensions of 15 mm × 5 mm × 1 mm were prepared from the PF1 and PF2 foams, obtaining 8 matrices. Immobilization of drugs (NMS, DOX, ALA, MOP) was carried out for each composition by incubating the matrices in freshly prepared drug solutions (1 mM; solvents were used as written above). After 24 h incubation at 4 °C, the matrices were removed from the drug solutions, washed with water and dried under reduced pressure at ambient temperature (Table 2, rows 1–4).

#### 2.4.3. Immobilization of Drugs for Cell Culture Study

The 40 pieces of foam (1 cm × 1 cm × 0.5 cm dimensions) were prepared from the PF1 foam (composition G3:G441) and PF2 (composition G3:G441:CO) and were grouped into samples as described in Table 2. Four samples of each foam (Table 2) were subjected to encapsulation of doxorubicin (DOX), and one for each kind of foam was left as reference sample. Drug immobilization was carried out at 4 °C for 24 h (1 mM concentration). After immobilization, the samples were washed and dried under reduced pressure at ambient temperature and radiation sterilized.

### 2.5. Cell Culture

Human squamous carcinoma cells SCC-15, provided by American Type culture Collection (ATCC, Manassas, VA, USA), were cultured in DMEMF12 with 400 ng/mL hydrocortisone and human immortalized keratinocytes HaCaT obtained from Cell Line Service (CLS, Germany) in DMEM medium, both supplemented with 10% heat-inactivated FBS, 100 U/mL penicillin and 100 μg/mL streptomycin. Cells were incubated at 37 °C in an atmosphere of 5% CO_2_ and 95% humidity. Medium was changed every 2–3 days, and cells were passaged at about 80% confluence with 0.25% trypsin–0.03% EDTA in calcium- and magnesium-free sterile PBS. Cell morphology was checked with the Nikon TE2000S inverted microscope (Tokyo, Japan) equipped with phase contrast. The number and viability of cells were estimated by the trypan blue assay with Automatic Cell Counter TC20™ (Bio-Rad Laboratories, Hercules, CA, USA).

### 2.6. Direct Contact Cytotoxicity Assay

Cytotoxicity of PAMAM G3 dendrimer-based foams was studied with direct-contact toxicity assay as described [59]. Squamous carcinoma cells (SCC-15) and immortalized keratinocytes (HaCaT) were seeded into 6-well plates in the amount of 8 × 10^5^ cells/well and incubated for 24 h in 37 °C, 5% CO_2_, 95% humidity. After that, medium was removed, and cells were washed with 1 mL of fresh complete medium per well. Sterile foam samples (about 10 × 10 mm, 5 mm thickness) were placed on cell monolayers and incubated for 24 h. Then, samples were removed, and cells were washed once with PBS and stained with 0.2% crystal violet in 2% ethanol for 30 min. After washing with distilled water (three times), images of plates with stained cells were collected, and the reactivity zones were assessed with ImageJ 1.49v. Obtained results were interpreted by the grade of the reactivity zone described by U.S. Pharmacopeial Convention [60] and ISO 10993-12 [61]. Solubilization of stained cells was performed with 10% acetic acid on shaker (1 mL/well, 10 min, 400 rpm, room temperature). The absorbance of CV solutions from the individual samples was measured at 595 nm against 450 nm and against a blank sample (10% acetic acid) with microplate reader (μQuantTM, BioTekInstruments, Inc., Winooski, VT, USA). Results were presented as a % of control. Three independent experiments were performed in triplicate.

### 2.7. Extract Cytotoxicity Assay with Neutral Red NR and XTT

The extracts were prepared in accordance with ISO 10993-12 (2012) guidelines in complete culture medium. Each foam (0.5 g/10 mL) was totally immersed in extraction medium and incubated at 37 °C for 24 h in static conditions. Extracts were sterilized with syringe filters (0.22 μm) and diluted in complete medium to 3–100% concentrations. HaCaT and SCC-15 cells were seeded into 96-well plates (1 × 10^4^ cells/well) and incubated 24 h in 37 °C. Then, medium was replaced with obtained extracts (100 μL/well), and cells were incubated for 24 h (37 °C, 5% CO_2_). As a control, cells without extracts were used. After 24 h exposure to extracts, medium was replaced by 2% neutral red in the culture medium (100 μL/well), and cells were incubated for 1 h. After rinsing with PBS, 100 μL/well of fixative (50% ethanol, 49% H_2_O and 1% glacial acetic acid) was added, and plates were shaken until complete dye dissolution (300 rpm., 15 min., room temperature). Absorbance was measured at 540 and 620 nm with a microtiter plate reader (μQuant–BioTek, Winooski, VT, USA) against a blank sample (fixative without cells). The XTT assay was performed as described [62]. Percentage cell viability was calculated by normalization of the absorbance readings against that of the non-treated cells (set as 100%). Three independent experiments were performed in triplicate.

### 2.8. Cellular Uptake of Doxorubicin Released Form Foams

HaCaT and SCC-15 cells were seeded into flat-bottom 96-well plates, 1 × 10^4^ cells per well, and allowed to achieve adhesion for 24 h at 37 °C. Then, culture medium was exchanged for extracts originating from foams previously soaked with doxorubicin (PF1-DOX/PF2-DOX), as described in Section 2.4.3. The control was an extract of the foams PF1 or PF2 without DOX. The extracts were used in a range of increasing concentrations (3.125–100%) and incubated with the cells for 24 h at 37 °C. Uptake of doxorubicin by cells was observed with Delta Optical IB-100 fluorescence microscope (Nowe Osiny, Poland) at an excitation light length of 480 nm and emission light of 590 nm.

### 2.9. Statistical Analysis

To estimate the differences between treated and non-treated control samples, statistical analysis was performed using the non-parametric Kruskal–Wallis test due to the lack of a normal distribution of data in the studied groups (analyzed with Shapiro–Wilk test). All analyses, calculations and figures were performed with Statistica 13.3 software (StatSoft, Cracow, Poland).

## 3. Results and Discussion

### 3.1. PAMAM G3 Dendrimer Foaming Procedure

Our goal was to obtain a porous material with a third-generation poly(amidoamine) dendrimer (PAMAM G3). We attempted to react PAMAM G3 with diisocyanate (pMDI), but these two reagents were not miscible, and further foaming with the addition of water and surfactant did not occur (Table 1, entries 1 and 2). Therefore, we decided to adopt a known procedure to obtain poyurethane foam based on polyol and pMDI. We chose the polyether polyol with a hydroxyl number in the range of 330–360 mg KOH/g and dynamic viscosity in the range of 250–310 mPa·s at 25 °C (Rokopol G441), which was miscible with G3 and was suitable as a liquefaction agent. After obtaining the reference foam from these substrates (Table 1, entry 3), we tested the foaming of the mixture containing PAMAM G3 dendrimer and G441 polyol with pMDI, water and surfactant (Table 1, entries 4 and 5). After mixing 50% wt G3 with 50% wt G441 at 50 °C and bringing it to room temperature, the G3:G441 mixture had the resin consistency. As it turned out, the resting time of the G3:G441 mixture before the foaming process, which was at least 24 h, also influenced the subsequent homogenization. When the problem with the consistency of G3 was solved, numerous tests were carried out to optimize the composition and develop a procedure to obtain PAMAM G3 foams (Table 1, entries 5–9). The optimized amount of water, surfactant and pMDI was settled as those for F1 reference. The PUFs obtained according to this protocol were irreproducible, mostly due to the fact that foam growth started before the ingredients were completely mixed. Therefore, the order of adding pMDI and the catalyst was changed compared to the standard procedure. This allowed the ingredients to be creamed and the foam to rise appropriately (Table 1, entry 5, marked as PF1). 

There was a need to use an additional ingredient that would improve the homogenization of the dendrimer with the remaining ingredients of the composition and influence regular growth and structure. Castor oil (CO) was used because it is a natural polyol and can be used in reactions with diisocyanates [63,64] without functionalization [65]. Castor oil itself did not liquefy the dendrimer well, and an appropriate foam was not obtained from mixture G3:CO (Table 1, entry 9). Therefore, the next step was to select appropriate amounts of polyfunctional ingredients in the G3:G441:CO mixture. As before, optimization began with the preparation of the F2 reference foam (Table 1, entry 10). Equivalent quantities of G441 and CO were used, as well as amounts of blowing agent, surfactant and catalyst determined for the PF1 foam. Leaving the amount of G3 as in the case of the PF1 foam (at 50% wt), a G3:G441:CO mixture was prepared with a CO content of 25, 15, and 5% (Table 1, entries 11–13). A 25% castor oil content led to poor growth and hard foam. With a 5% CO content, the growth was sufficient, and a semi-rigid but fragile foam was obtained. The use of 15% castor oil resulted in a semi-rigid, non-fragile foam with a visually homogenous structure (Table 1, entry 12, marked as PF2).

### 3.2. Properties of PAMAM G3 Dendrimer-Based Foams

The apparent density of the PF1 and PF2 foams was determined and was found to be 51.4 kg/m^3^ in the case of PF1 and 32.6 kg/m^3^ in the case of PF2 (Table 3). 

Both foams had a relatively low apparent density, which is within the density range of both rigid and flexible foams. The obtained foams are characterized by relatively high water absorption, with the highest absorption achieved after 24 h of exposure of the samples to water (Table 3). PF1 has almost twice as large water absorption as PF2. These results indicated that the foam cells are open in both materials.

### 3.3. The Morphology of PAMAM G3 Dendrimer-Based Foams

SEM analysis of PF1 and PF2 foams and their F1 and F2 standards showed that all foams are composed of thin ribs/walls forming an interconnected network containing hollow bubbles/pores. The reference foam F1 obtained by foaming Rokopol G441 is characterized by a relatively regular structure with average pore sizes equal to 590.66 μm. The foams PF1 obtained from the PAMAM G3:G441 composition have a structure similar to the standard sample F1 but of a more irregular nature. They have pores with an average 526.75 μm diameter. The large dispersion of pore sizes is the result of insufficient homogenization during foaming and the presence of dendrimer clusters in the foam structure.

**Figure 1:**
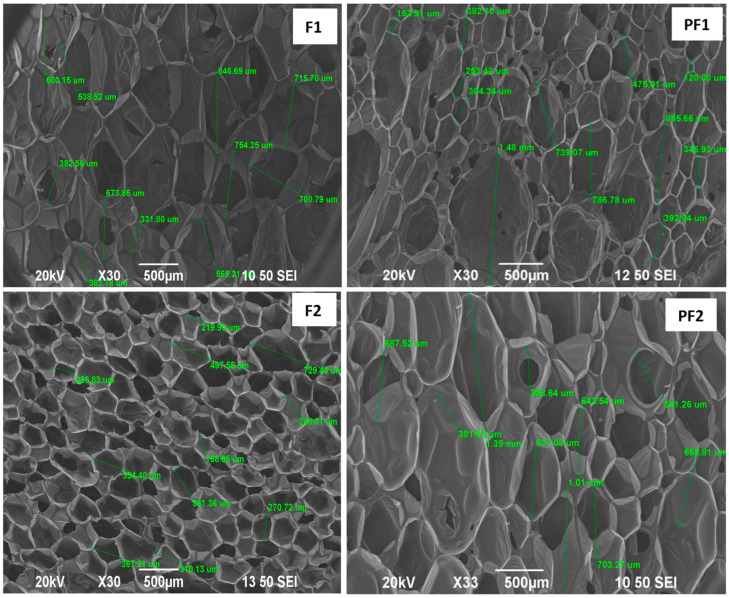
SEM micrographs for standard foam F1 (obtaining from G441); F2 (obtaining from G441:CO mixture); PF1, PF2 containing PAMAM G3 additionally.

The reference foam F2 obtained by foaming Rokopol G441 with the addition of CO is characterized by a more regular structure and smaller pores (most of the order of 200–400 μm, single pores size 700 μm) than the reference foam F1 (without CO). The foam PF2 obtained from the PAMAM G3, Rokopol G441 and CO composition have a more irregular structure and larger pores (400 to 1400 μm) with a larger size distribution compared to the standard sample F2 (Table 4, Figure 1).

**Figure 2:**
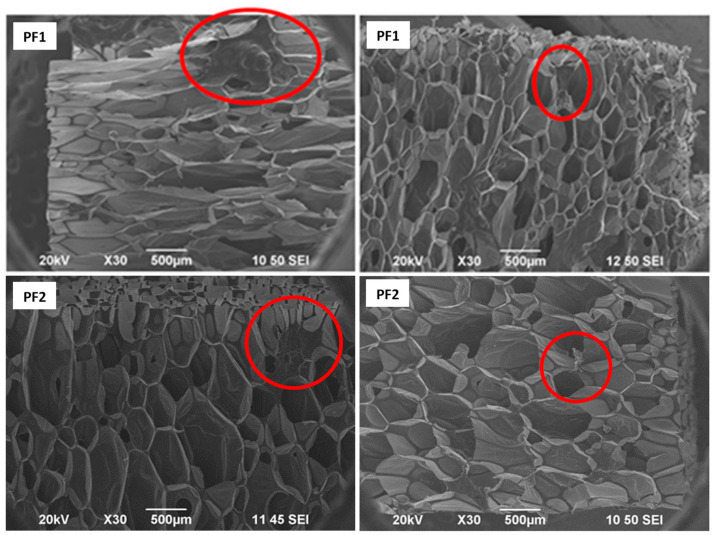
SEM images illustrating pore defects in foam PF1 (up) and PF2 (down). Red circles show irregularities in the structure.

Compared to the reference foams F1 and F2, both foams with PAMAM G3 show some irregularities in the structure. In the case of PF1, defects are visible in the form of thickenings and polymer clusters with sizes of several hundred micrometers. PF2 also shows defects in the structure, but this foam is characterized by a more regular structure than samples from the composition without castor oil PF1 (Figure 2).

### 3.4. Drug Encapsulation Efficiency in the PF1 and PF2 Matrices

**Figure 3:**
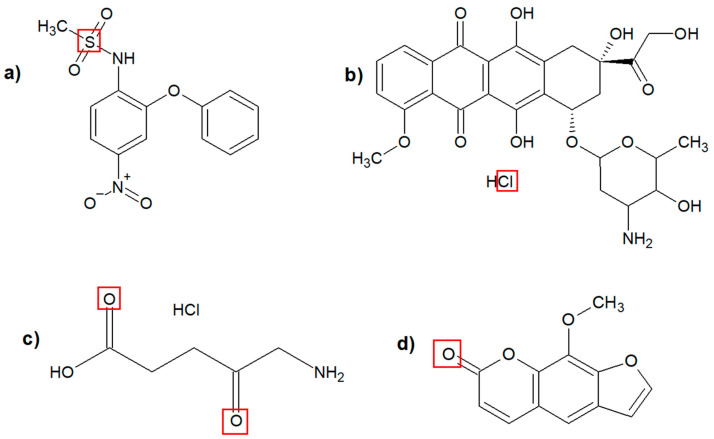
Structure of drugs used for immobilization in matrices PF1 and PF2: (**a**) nimesulide, (**b**) doxorubicin hydrochloride, (**c**) aminolevulinic acid hydrochloride, (**d**) 8-methoxypsoralen. Red squares indicated chemical moieties specific to the corresponding drugs.

In order to assess the ability of PF1 and PF2 foams to bind drugs, the foam surfaces after encapsulation of four different drugs (Table 2, rows 1–4) were tested using the XPS method. The binding efficiency of drugs in the matrices was estimated based on the content of chemical moieties specific to the corresponding drugs (Figure 3). 

Nimesulide content was estimated based on the S 2p peak located at 169 eV (Figure S1A,B), doxorubicin hydrochloride based on the Cl 2p peak at 197 eV (Figure S1C,D), aminolevulinic acid hydrochloride and 8-methoxypsoralen were calculated based on carbonyl oxygen content other than polyurethane (PU) and polyurea (PUa) O1s peak located at 532 eV (Figure S1E,F). The subsequent calculations allowed us to determine the mass percentage of drugs in the PF1 and PF2 matrices (Table 5). 

The drug-binding efficiency of individual matrices varied depending on the drug. In the case of NMS and MOP, the PF2 matrix binds drugs more effectively. This can be attributed to the more hydrophobic properties of PF2, which is consistent with the twice-larger water uptake of PF1 compared to that of PF2 (Table 1). Thus, less hydrophilic PF1 was able to absorb twice less NMS or MOP than PF2. On the contrary, more ALA and DOX hydrochlorides were absorbed in hydrophilic PF1 in comparison with PF2. Considering the percentage of DOX in PF2 and the apparent density of this PUF, the samples used later in biological tests contained ca 0.25 mg DOX per 0.5 cm^3^ (430 nmoles load of DOX).

### 3.5. Cytotoxicity

The PAMAM dendrimers’ cytotoxicity depends strongly on the number and nature of functional surface groups. Cationic dendrimers exhibit rather high toxicity due to their interaction with negatively charged cell membranes. Dendrimers are intrinsically toxic, thus creating a major limitation for their use in biological systems. The reduction in toxicity may be achieved through their surface engineering [66]. Additionally, PAMAM dendrimers administered intravenously cause numerous side effects in vivo, such as immunotoxicity, hemolytic toxicity, neurotoxicity, gastrointestinal toxicity, hepatorenal toxicity, nephrotoxicity, pulmonary toxicity and cardiotoxicity [67]. Therefore, in order to avoid systemic side effects, we proposed local therapy using dendrimer matrices capable of releasing and more efficient delivery of drugs in diseased areas.

The obtained dendrimer matrices may be used as drug delivery devices with the ability to release drugs, for instance, in cancer therapy. Such matrices should be biocompatible and, at the same time, demonstrate the ability to immobilize and then release bounded drugs. In order to test the biocompatibility of the obtained foams and matrices, in vitro cytotoxicity tests were performed: direct contact assay and extract assay according to ISO and US Pharmacopeia standards.

**Figure 4:**
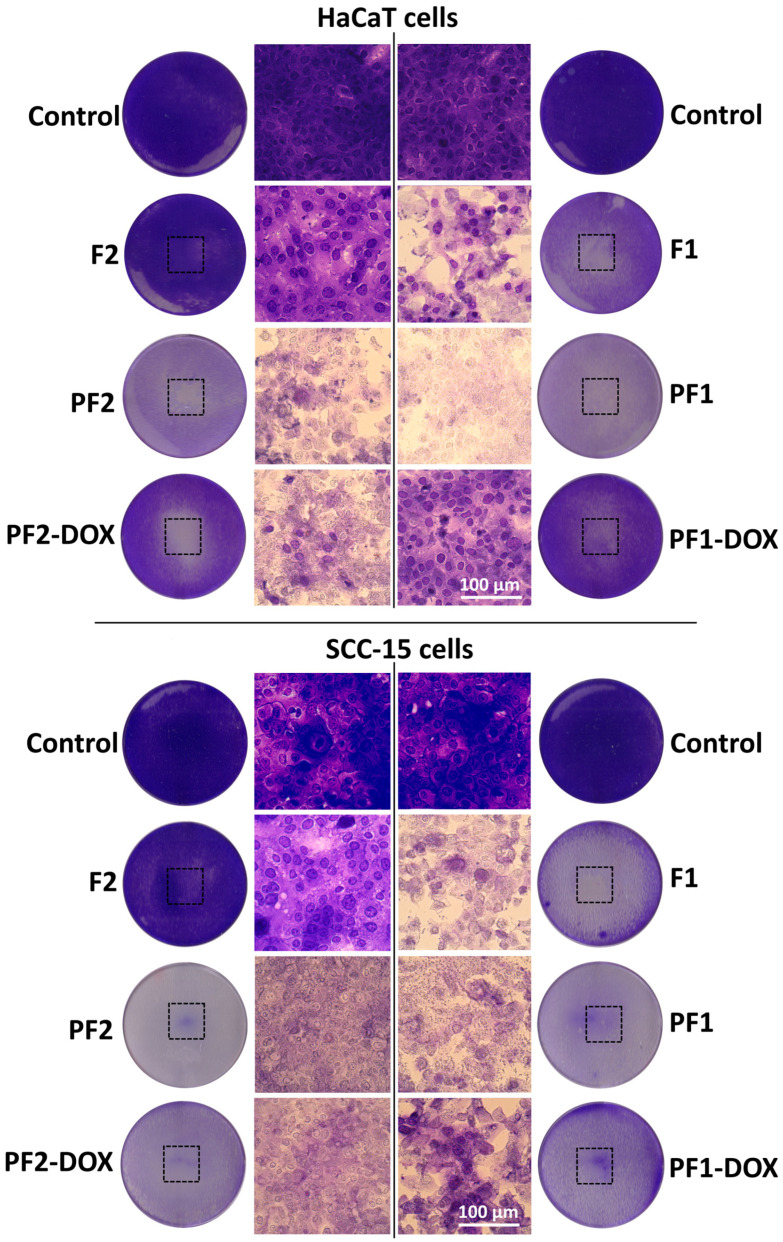
Microscopic images of human immortalized keratinocytes (HaCaT) and human squamous cell carcinoma cells (SCC-15) stained with crystal violet after 24 h incubation with the studied PAMAM G3 dendrimer-based foams. Round images show the plate wells containing stained cells with black squares indicating the localization of the samples. The medial rows show cell morphology from central parts of reactivity zones.

**Figure 5:**
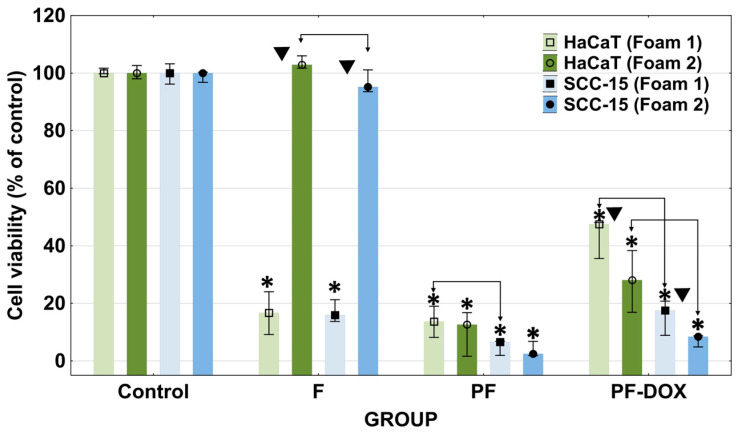
The viability of human immortalized keratinocytes (HaCaT) and human squamous cell carcinoma cells (SCC-15) after 24 h incubation with dendrimer-based foam samples. Results obtained after direct contact cytotoxicity assay are expressed as medians of nine measurements from three independent experiments. The lower (25%) and upper (75%) quartile ranges are presented as whiskers. Asterisk * indicates differences between control and samples (*p* < 0.05, Kruskal–Wallis test). Symbol ▼ means significant differences between different foams for corresponding cell lines (*p* < 0.05, Mann–Whitney U test). Arrows indicate differences between the response of HaCaTs and SCC-15 cells for appropriate foam.

Direct contact assay indicated that the most biocompatible was F2 foam containing castor oil. After 24 h of incubation, it did not significantly affect the viability of HaCaT epidermal cells or SCC-15 squamous cell carcinoma cells and did not cause damage to these cells in the area covered by the sample (Figure 4 and Figure 5). Meanwhile, the F1 foam, which did not contain castor oil, showed significant toxicity towards both tested cell lines, causing a decrease in their viability to below 20% compared to the untreated control (Figure 4). Microscopic images showed that in HaCaT cells reactivity zone was limited to the area under the specimen with some malformed or degenerated cells (slight reactivity), but in SCC-15 cells reactivity zone extended 0.45–1.0 cm beyond the specimen (moderate reactivity) (Figure 4).

**Figure 6:**
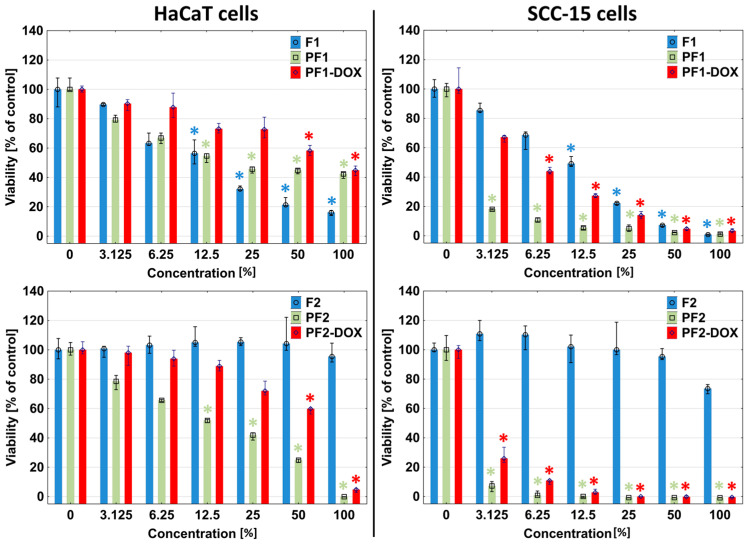
Response of HaCaT and SCC-15 cells for extracts from studied matrices after 24 h incubation estimated with NR assay. Results are expressed as % of non-treated control (control = 100% viability). The lower (25%) and upper (75%) quartile ranges are presented as whiskers. Asterisk * indicates statistically significant differences between non-treated control and samples (*p* < 0.05, Kruskal–Wallis test).

**Figure 7:**
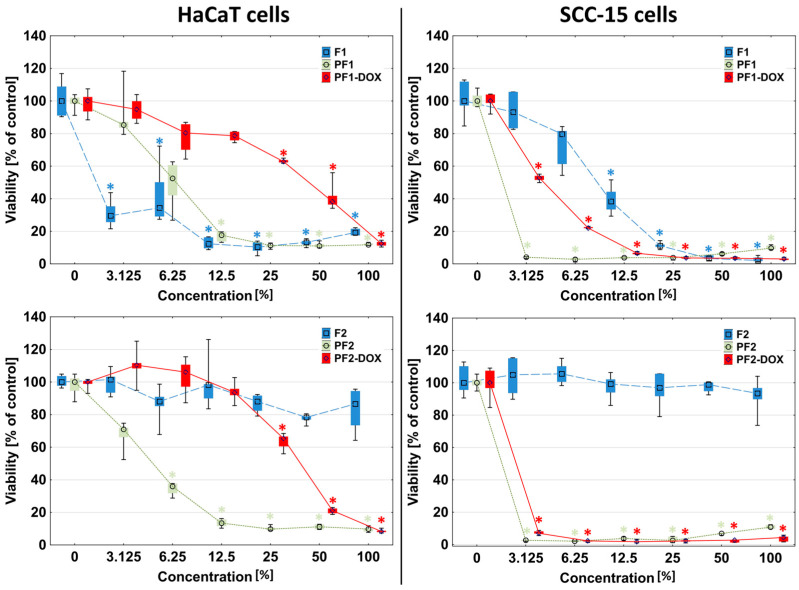
Sensitivity of HaCaT and SCC-15 cells on F1, F2, PF1, PF2, PF1-DOX and PF2-DOX extracts after 24 h incubation estimated with XTT assay. Results are medians expressed as % of non-treated control. The lower (25%) and upper (75%) quartile ranges are presented as whiskers. Asterisk * indicates statistically significant differences between non-treated control and samples (*p* < 0.05, Kruskal–Wallis test).

Analogous results were obtained in extract assays with neutral red (NR) and tetrazolium salts (XTT). Moreover, 100% F1 foam extracts reduced the viability of HaCaT cells to approximately 20% and the viability of SCC-15 tumor cells to near zero (Figure 6 and Figure 7). The first signs of significant toxic effects were visible against both cell types from 12.5% extract concentration (NR test). The XTT assay showed that HaCaT cells responded significantly for 3.125% F1 extract and SCC-15 for 12.5%. F2 foam extract did not affect both cell types (NR and XTT).

XTT assay evaluates the reducing properties of trans-plasma membrane electron transport, including the activities of mitochondrial oxidoreductases, so it is an indicator of the mitochondrial condition [68]. The significantly greater sensitivity of HaCaT cells to F1 extract in the XTT assay than in other tests shows that substances contained in the F1 extract impaired the activity of mitochondria.

The most biocompatible was F2 foam with rocopol and castor oil (CO). Castor oil and rocopol were used to synthesize foams, which showed low cytotoxicity against cells [63,69]. Studies have attempted to determine half-maximal inhibitory concentration (IC_50_) values for polyurethane foams containing rocopol, but this was not possible because the cytotoxicity was too low, even at the highest concentrations. An IC_50_ was determined for only one of the foams, containing a 96.1% concentration of rocopol [69]. The most harmful ingredient used in the synthesis of polyurethane foams is isocyanates (pMDI). Foams synthesized from aromatic isocyanates in contact with the cell environment may degrade to toxic aromatic amines, so aliphatic isocyanates are the best choice [70,71]. The pMDI we used has toxic and mutagenic properties. The higher toxicity of F1 foam compared to F2 was probably due to the fact that F1 had a higher content of toxic pMDI (4.1 g) and G441 (4.1 g) compared to F2 (3.4 g pMDI and 2 g G441) (Table 1). The 50% of G441 in F2 was replaced by CO. However, polyurethane foams synthesized from polyols, which include rocopol and MDI, show near-zero cytotoxicity [72,73]. 

The addition of PAMAM G3 dendrimers to the F1 and F2 foams resulted in an increase in the toxicity of the obtained matrices, especially drastic in the case of F2 foam. Cells of both lines showed a strong reduction of viability after 24 h of incubation with PF1 and PF2 by more than 80%. The reactive zones covered almost the entire area of the plate well, and the cells in the area covered by the samples were shrunken and discolored, with a stronger effect visible in the case of squamous carcinoma cells (Figure 4 and Figure 5). Results obtained in NR and XTT assay were consistent with those observed in the direct contact assay, where at any extract concentration (3.125–100%), the presence of dendrimer contained in the foams (PF1 and PF2) increased the toxicity of the foams compared to foams without dendrimer (Figure 6). Only the PF1 extract at 25–100% concentration (NR assay) or 3–12.5% (XTT assay) was less toxic than the F1 foam against HaCaT cells (Figure 6). Moreover, the sensitivity of HaCaT cells on PF1 extract in the XTT assay was greater than in the NR assay, which again confirms the higher sensitivity of HaCaT cells’ mitochondria to substances contained in the F1 and PF1 extract. To better illustrate the differences in the toxicity of the tested foam extracts, the half-maximal inhibitory concentration (IC_50_) values are presented in Table 6.

PAMAM dendrimers are cationic polymers with terminal amine groups, which may exhibit toxic effects via interaction with negatively charged cell membranes or proteins in a cell and cause cell lysis. The generation number is crucial—the higher it is, the higher the toxicity [67]. Therefore, in this study, we used relatively low-toxic, third-generation PAMAM dendrimers to avoid cytotoxic effects. Unfortunately, too high a dendrimer content in the matrices is likely the reason for the high toxicity of the tested matrices. It is possible that the dendrimer was partially released from the tested matrices during incubation and was a factor increasing the toxicity of F2 due to poorer cross-linking of the dendrimer in F2 foam. The solution may be to reduce the dendrimer concentration or its modification by glycolation [75], vitamin conjugation [76], acylation [77] and PEGylation [78]. Dendrimers with hydroxyl surface groups do not exhibit toxicity, and additionally, they can become a reagent during the creation of dendrimer polyurethane matrices. This issue will be the subject of our further research.

Furthermore, for the synthesis of PF2 foam, the necessary amount of pMDI was calculated with respect to the number of G441 and CO hydroxyl residues and surface amino residues of the dendrimer (32 per dendrimer molecule). Due to the inhomogeneous distribution of PAMAM G3 and steric hindrance on the surface of its molecules, a large part of the pMDI could not react and be responsible for the high toxicity of PF2. This effect was not observed in the case of the PF1 matrix due to the high toxicity of F1. 

Doxorubicin (DOX) is a hydrophobic, cytostatic agent commonly used in the therapy of breast, lung, leukemia, brain and lymphoma [79]. It can be immobilized inside PAMAM dendrimer cavities [80,81] or linked to the surface of modified amino groups [51,82]. In these studies, DOX was immobilized via encapsulation using the obtained PF1 and PF2 dendrimer matrices. We expected that immobilization of DOX by encapsulation would enhance the toxicity of the matrices since it is known that DOX is active at very low nanomolar concentrations [83,84,85]. Meanwhile, matrices containing DOX and their extracts showed significantly less toxicity than PF1 and PF2 matrices alone in all performed direct contact and extract assays (Figure 4, Figure 5, Figure 6 and Figure 7). The immobilization of DOX with the PF1 matrix (PF1-DOX) resulted in a 2.5-fold increase in the viability of HaCaT cells compared to PF1 and, in the case of SCC-15 cells, less than two times. In the case of PF2 and PF2-DOX, this increase was no more than two times (Figure 5). Similar results were described by Szota et al. [86]. Handriela Hoff de Oliveira Sobrinho et al. described the interactions that occur in a nanoparticulate system with the potential for a controlled drug release using density functional theory, as implemented in the SIESTA code. The results showed the presence of a hydrogen bonding interaction between PAMAM and doxorubicin [87]. Adsorption of doxorubicin on a PAMAM G3 dendrimer could, therefore, contribute to reducing the toxicity of the dendrimer released from the tested matrices. The reaction of DOX and amino groups of PAMAM G3 was impossible due to the encapsulation of the foams at a temperature of 4 °C.

### 3.6. Cellular Uptake of Doxorubicin Released from Foams

The encapsulated drugs should be able to be released from the carriers to ensure the therapeutic effect. Therefore, we studied the in vitro release of DOX from dendrimeric matrices in DMEM (for HaCaT cells) or DMEMF12 (for SCC-15 cells) medium. After 24 h incubation, DOX released from dendrimeric matrices PF1-DOX and PF2-DOX was taken up by squamous cell carcinoma SCC-15 cells and human immortalized skin keratinocytes HaCaT cells. 

**Figure 8:**
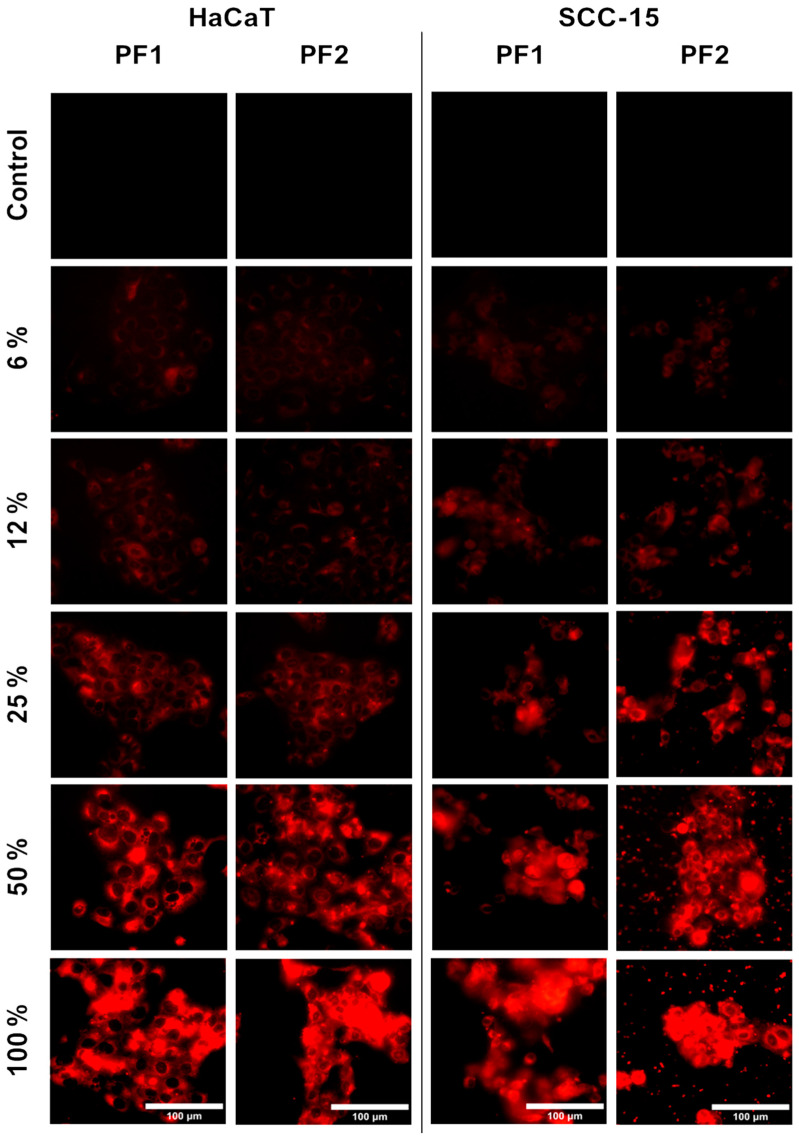
Uptake of doxorubicin released from PF1 and PF2 foams by HaCaT and SCC-15 cells after 24 h incubation. Images were collected with IB-100 Delta Optical fluorescence microscope at 480/590 nm excitation/emission wavelength.

DOX accumulation in cells was proportional to extract concentration. Furthermore, microscopic images show that the fluorescent signal from DOX appeared mainly in the cytoplasm. As shown in other studies, DOX accumulates mainly in cell nuclei and in trace amounts in the cytoplasm [88,89]. The appearance of a red signal from DOX in the cytoplasm but not nuclei suggests the release of the dendrimer from the PF1 and PF2 matrices together with the encapsulated DOX (Figure 8). This phenomenon was also observed by others: PAMAM dendrimers conjugated with DOX accumulated mainly in the cytoplasm [53,90]. This confirms the usefulness of dendrimer matrices in the immobilization and release of drugs into normal and cancer cells. 

In addition to PAMAM dendrimers, micelles and liposomes are also being investigated for DOX-targeted transport [91]; however, each of these nanocarriers has some limitations. Liposomes exhibit the poor release of DOX and significant accumulation in lysosomes [92], which limits delivery to the nucleus [93]. Micelles, in turn, are characterized by low stability, leading to the release of encapsulated drugs before reaching the target site, which also requires appropriate modifications of the carrier [94]. The use of native PAMAM dendrimers as vehicles for DOX in targeted therapy is known and tested [95]. DOX encapsulation in PAMAM carrier cavities in a polymer matrix further outweighs the benefits of being able to use a free, native PAMAM dendrimer as its vehicle. Additionally, studies of DOX release from PF1 and PF2 matrices and uptake by HaCaT and SCC-15 cells also provide the potential for use in transdermal transport of other drugs tested in this study (Nimesulide, 8-Methoxypsolarene, 5-Aminolevulinic acid) for which the ability of the matrices to their immobilization was demonstrated.

## 4. Conclusions

Our studies show that PAMAM dendrimers can be a valuable component, creating dendrimer matrices based on rocopol and castor oil. The PF2 foam had much better properties than PF1, showing very high toxicity mainly towards SCC-15 cancer cells, but at the same time, it efficiently released a model anticancer drug—doxorubicin encapsulated in the PAMAM G3 dendrimer, probably together with the dendrimer. Therefore, the obtained results suggest that the studied materials can be used as matrices for the controlled release of drugs and biologically active agents for anticancer therapy. Hydrophobic drugs can easily encapsulate in the cavities of PAMAM dendrimer molecules and can be released from them as native drugs or in complexes with the released PAMAM dendrimer molecules, which means that they might penetrate into adjacent tissues even more efficiently than native drugs, also through the epidermal barrier. Dendrimer matrices would be placed by contact in places where cancer cells are present, also after tumor resection. Their use as carriers of repositioned drugs for cancer therapy, alleviating inflammation associated with cancer (celecoxib, nimesulide) and enabling PUVA therapy (8-methoxypsoralen, aminolevulinic acid) cannot be ruled out. Since PAMAM dendrimers can be excellent vehicles for nucleic acid-based therapeutic agents, such as antisense oligonucleotides, ribozymes, siRNA and plasmid DNA in human cells, dendrimeric matrices can also be used for this purpose. 

The next step will be the reduction of the toxicity of the dendrimer contained in the matrix, for instance, by partial hydroxylation of the surface dendrimer residues and optimization of its content in foaming composition.

## Figures and Tables

**Table 1:** The composition of foams based on PAMAM G3 dendrimer (G3), Rokopol G441, castor oil (CO) and the foaming process.

Foam Number/ Name	Substrate Ratio G3:G441:CO [wt%]	Composition							Foaming Process			Characteristics of Freshly Prepared Foams
		G3 [g]	G441 [g]	CO [g]	pMDI [g]	H_2_O [g]	Silicone [g]	TEA [g]	Time of Creaming [s]	Time of Expanding [s]	Time of Drying [min]	
1.	G3	2	-	-	1.43	0.04	0.054	0.034	-	-	-	no foaming
	100											
2.	G3	2	-	-	1.43	0.08	0.054	0.034				
	100											
3. F1	G441	-	4	-	4.1	0.16	0.160	0.068				reference polyurethane foam
	100											
4.	G3:G441	2.4	1.6	-	3.7	0.16	0.160	0.068	-	-	-	low homogenization heterogeneous structure
	60:40											
5. PF1	G3:G441	2	2	-	3.8	0.16	0.160	0.068	20	90	60	small shrink, irregular pores
	50:50											
6.	G3:G441	2	2	-	3.8	0.08	0.160	0.068	22	60	60	insufficiently grown, hard
	50:50											
7.	G3:G441	2	2	-	3.8	0.16	0.213	0.068	19	90	60	small shrink, irregular pores
	50: 50											
8.	G3:G441	2	2	-	3.8	0.16	0.160	0.102	17	30	-	cracked, fragile
	50:50											
9.	G3:CO	2	-	2	3.1	0.16	0.160	0.068	-	-	-	ungrown, hard
	50:50											
10. F2	G441:CO	-	2	2	3.4	0.16	0.160	0.068				reference polyurethane foam
	50:50											
11.	G3:G441:CO	2	1	1	3.4	0.16	0.160	0.068	15	100	150	insufficiently grown
	50:25:25											
12. PF2	G3:G441:CO	2	1.4	0.6	3.56	0.16	0.160	0.068	15	120	150	semi-rigid, small, regular pores
	50:35:15											
13.	G3:G441:CO	2	1.8	0.2	3.7	0.16	0.160	0.068	18	110	150	semi-rigid, fragile
	50:45:5											

**Table 2:** Foam abbreviations after drug immobilization.

No	Immobilized Drug	Foam Composition	
		G3:G441	G3:G441:CO
1	nimesulide	PF1-NMS	PF2-NMS
2	doxorubicin hydrochloride	PF1-DOX	PF2-DOX
3	aminolevulinic acid hydrochloride	PF1-ALA	PF2-ALA
4	8-methoxypsoralen	PF1-MOP	PF2-MOP
5	none	PF1	PF2

**Table 3:** Foams PF1 and PF2 apparent density and water absorption.

Foams Composition	Apparent Density [kg/m^3^]	Water Absorption [%]			
		After 5 min	After 1 h	After 3 h	After 24 h
G3:G441	51.4	10.8	13.7	16.6	19.3
G3:G441:CO	32.6	4.1	7.0	7.3	10.5

**Table 4:** Pore sizes of F1, F2, PF1 and PF2 foams were calculated using SEM images.

Sample	Pore Sizes [μm]	
	Mean	Standard Deviation
F1	590.66	160.51
PF1	526.75	371.10
F2	344.93	147.99
PF2	705.10	283.52

**Table 5:** Drug content in matrices estimated based on XPS analysis.

Matrix	Diagnostic Peak/Moiety	Binding Energy [eV]	Peak/Moiety Content [%]	Drug Content [%]
PF1-NMS	S 2p	169.05	0.2	1.92
PF2-NMS		169.39	0.3	2.89
PF1-DOX	Cl 2p	197.14	0.4	6.5
PF2-DOX		197.91	0.1	1.63
PF1-ALA	O1s/C=O (other than PU and Pua *)	532.46/531.25	17.8/8.6 = 1.53	8.3
PF2-ALA		532.44/531.17	16.1/6.8 = 1.09	5.72
PF1-MOP		531.87/531.21	20.4/3.1 = 0.63	8.5
PF2-MOP		532.17/531.18	15.5/6.6 = 1.02	13.8

* *PU*—*polyurethane*, *Pua*—*polyurea*.

**Table 6:** The half-maximal inhibitory concentration (IC_50_) values determined following 24 h treatment of HaCaT and SCC-15 cells with extracts of studied matrices with NR or XTT assay. The values of IC_50_ were calculated with AAT Bioquest IC_50_ calculator [74].

	IC_50_ [%] NR Assay		IC_50_ [%] XTT Assay	
Sample	HaCaT	SCC-15	HaCaT	SCC-15
F1	10.23	10.86	<3.13	10.07
PF1	26.39	<3.13	6.21	<3.13
PF1-DOX	76.77	5.10	34.48	3.11
F2	≫100.00	≫100.00	≫100.00	≫100
PF2	13.10	<3.13	4.47	<3.13
PF2-DOX	51.54	<3.13	29.61	<3.13

IC_50_ values for foam extracts containing PAMAM G3 dendrimer (PF1 and PF2) were rather low, which means that the potential effects of DOX might be masked by the toxic effect of matrices and non-specific toxicity against normal cells.

## Data Availability

The raw data supporting the conclusions of this article will be made available by the authors on request.

## Supplementary Materials

The following supporting information can be downloaded at: https://www.mdpi.com/article/10.3390/ma17163905/s1, Figure S1. A. Wide scan XPS spectra for PF1-NMS. B. Wide scan XPS spectra for PF2-NMS. C. Wide scan XPS spectra for PF1-DOX. D. Wide scan XPS spectra for PF2-DOX. E. Narrow scan XPS spectra of O 1s for PF1-ALA. F. Narrow scan XPS spectra of O 1s for PF2-ALA. G. Narrow scan XPS spectra of O 1s for PF1-MOP. H. Narrow scan XPS spectra of O 1s for PF2-MOP.
