# Supplementary material for: A Novel PAMAM G3 Dendrimer-Based Foam with Polyether Polyol and Castor Oil Components as Drug Delivery System into Cancer and Normal Cells
Source: Materials (Basel). 2024 Aug 7;17(16):3905. doi: 10.3390/ma17163905 (PMC11355831; doi:10.3390/ma17163905)
Supplement: Supplementary file 1 [file materials-17-03905-s001.zip › materials-3088425-supplementary.pdf]

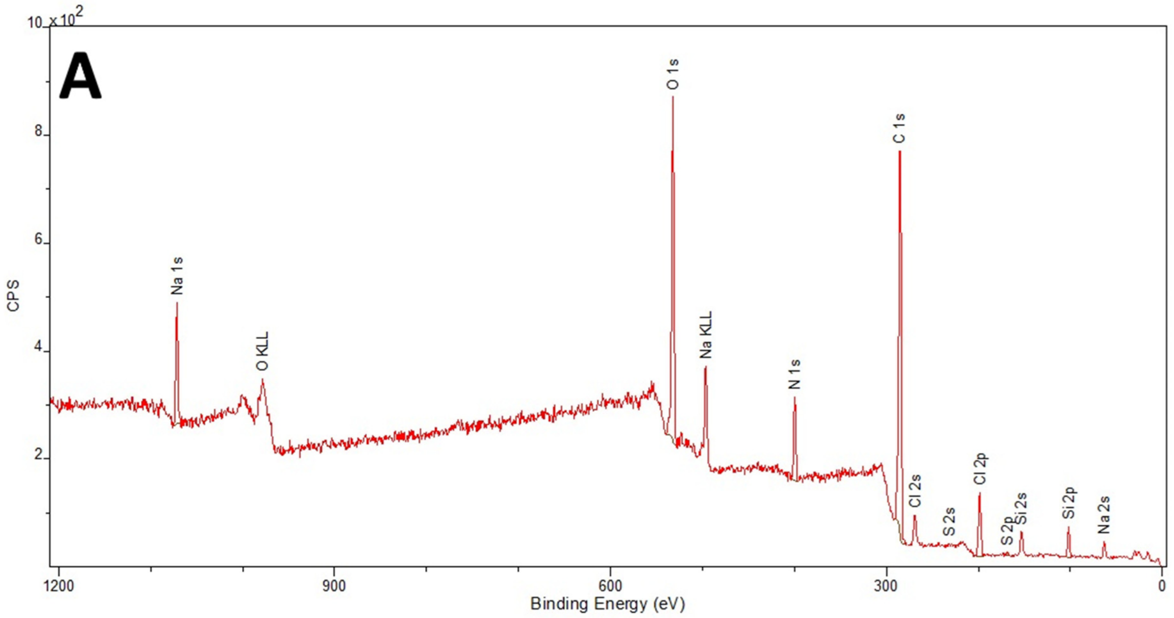

A
10 x10^2
8
6
4
2
CPS
Na 1s
O KLL
O 1s
Na KLL
N 1s
C 1s
Cl 2s
S 2s
Cl 2p
S 2p
Si 2s
Si 2p
Na 2s
1200
900
600
300
0
Binding Energy (eV)

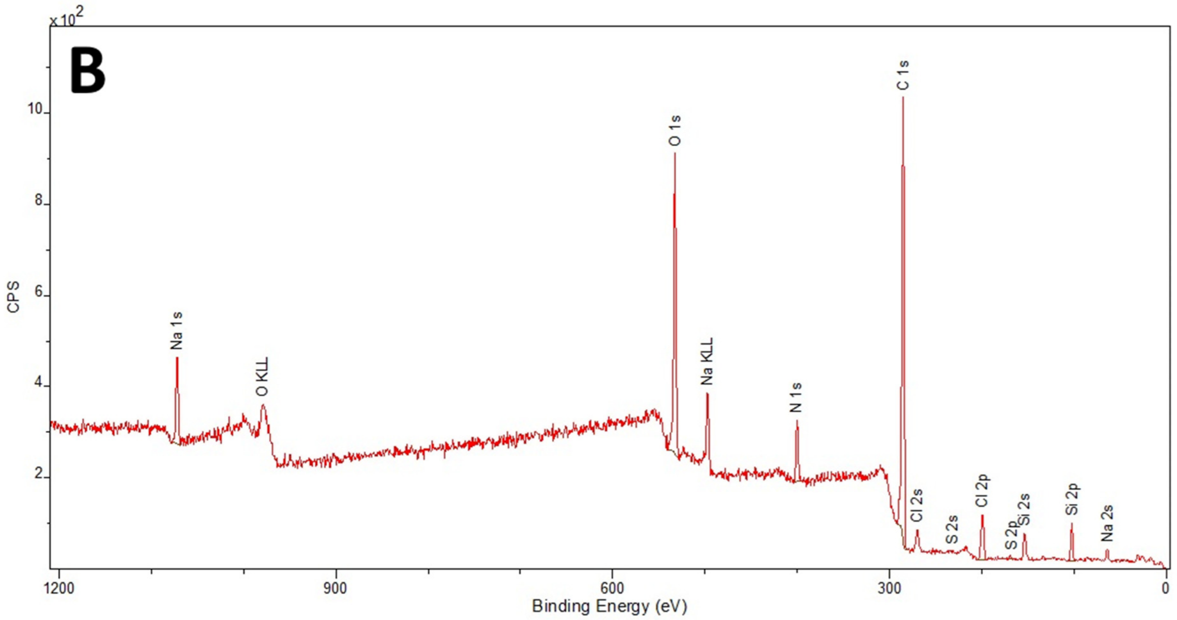

B
x10^2
10
8
6
4
2
CPS
Na 1s
O KLL
O 1s
Na KLL
N 1s
C 1s
Cl 2s
S 2s
Cl 2p
S 2p
Si 2s
Si 2p
Na 2s
1200
900
600
300
0
Binding Energy (eV)

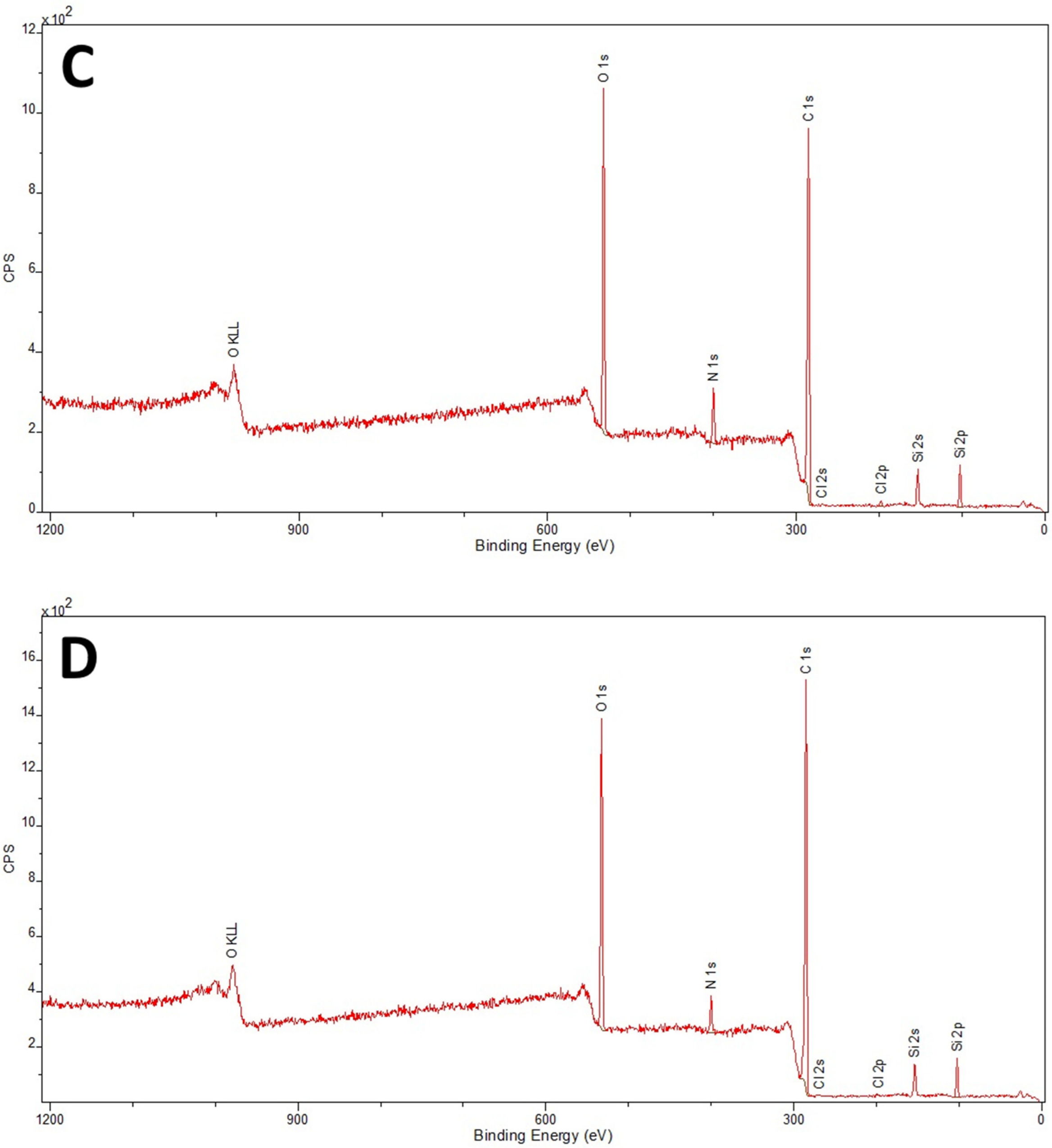
C
D
x10^2
CPS
Binding Energy (eV)
O KLL
O 1s
N 1s
C 1s
Cl 2s
Cl 2p
Si 2s
Si 2p
1200
900
600
300
0

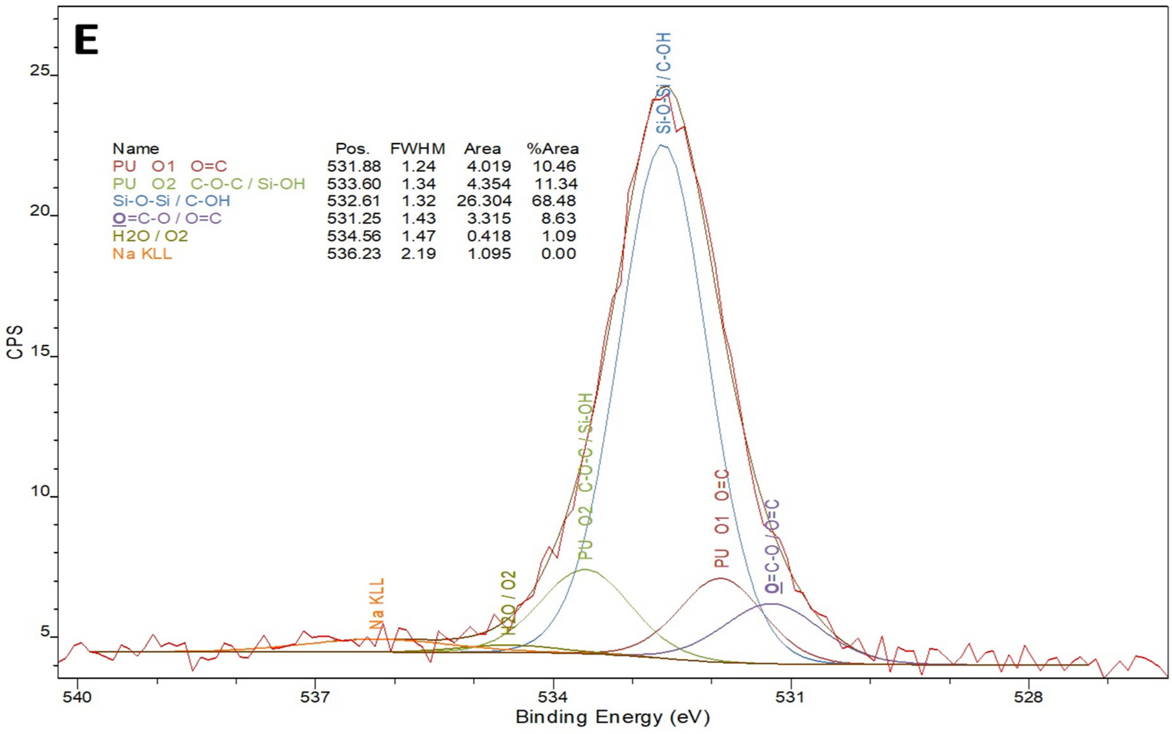
E
Name Pos. FWHM Area %Area
PU O1 O=C 531.88 1.24 4.019 10.46
PU O2 C-O-C / Si-OH 533.60 1.34 4.354 11.34
Si-O-Si / C-OH 532.61 1.32 26.304 68.48
O=C-O / O=C 531.25 1.43 3.315 8.63
H2O / O2 534.56 1.47 0.418 1.09
Na KLL 536.23 2.19 1.095 0.00
Si-O-Si / C-OH
PU O2 C-O-C / Si-OH
PU O1 O=C
O=C-O / O=C
H2O / O2
Na KLL
CPS
Binding Energy (eV)
25
20
15
10
5
540
537
534
531
528

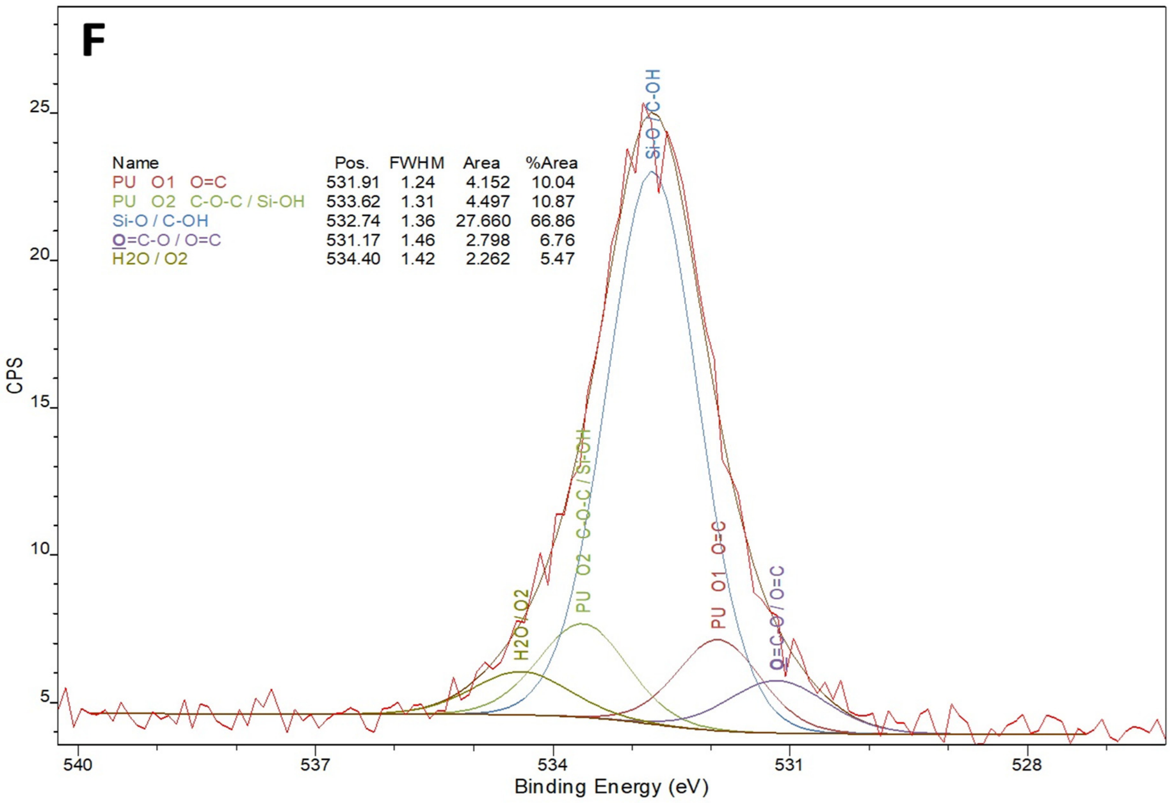
F
Name Pos. FWHM Area %Area
PU O1 O=C 531.91 1.24 4.152 10.04
PU O2 C-O-C / Si-OH 533.62 1.31 4.497 10.87
Si-O / C-OH 532.74 1.36 27.660 66.86
O=C-O / O=C 531.17 1.46 2.798 6.76
H2O / O2 534.40 1.42 2.262 5.47
Si-O / C-OH
PU O2 C-O-C / Si-OH
PU O1 O=C
O=C-O / O=C
H2O / O2
CPS
Binding Energy (eV)
25
20
15
10
5
540
537
534
531
528

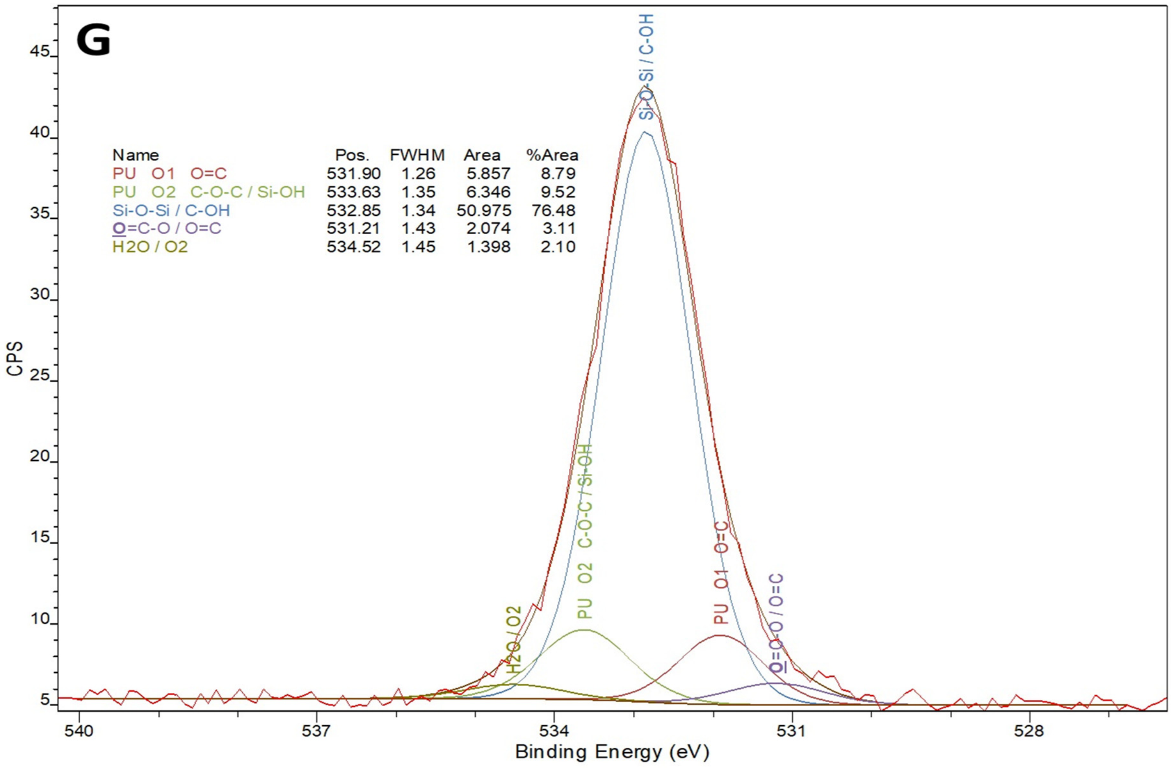

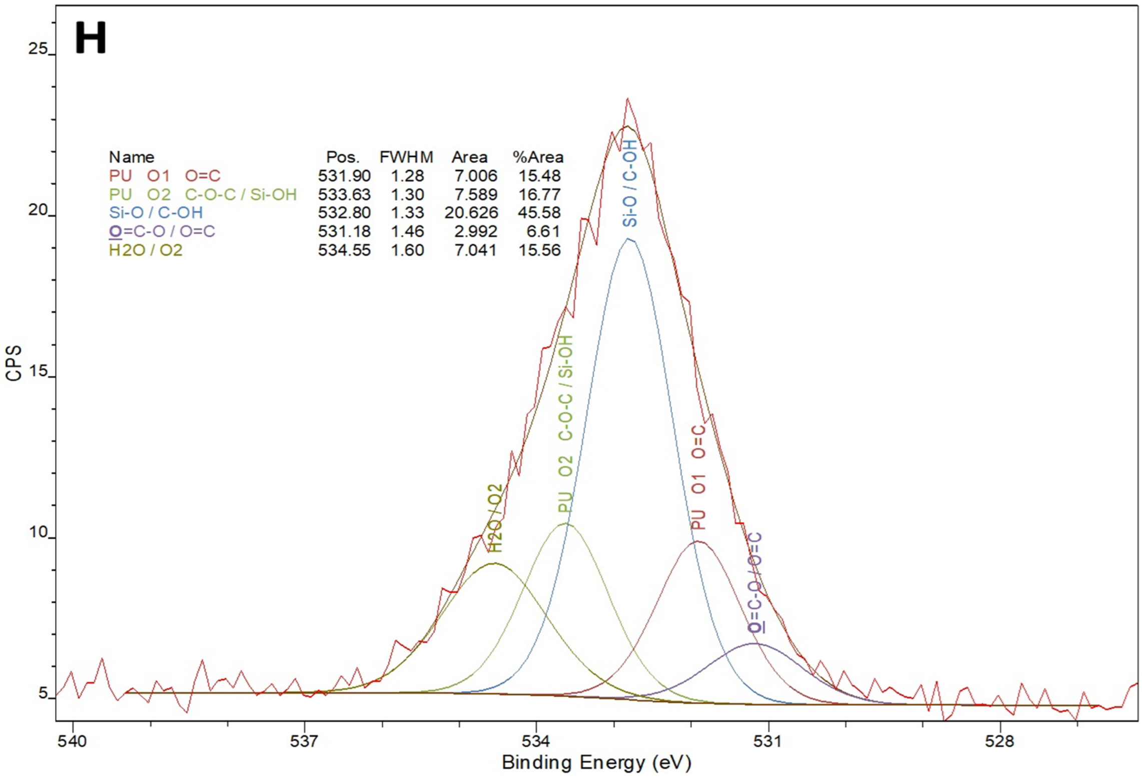

Figure S1. A. Wide scan XPS spectra for PF1-NMS. **B.** Wide scan XPS spectra for PF2-NMS. **C.** Wide scan XPS spectra for PF1-DOX. **D.** Wide scan XPS spectra for PF2-DOX. **E.** Narrow scan XPS spectra of O 1s for PF1-ALA. **F.** Narrow scan XPS spectra of O 1s for PF2-ALA. **G.** Narrow scan XPS spectra of O 1s for PF1-MOP. **H.** Narrow scan XPS spectra of O 1s for PF2-MOP.
